# Author Correction: p300 nucleocytoplasmic shuttling underlies mTORC1 hyperactivation in Hutchinson–Gilford progeria syndrome

**DOI:** 10.1038/s41556-025-01850-3

**Published:** 2025-11-28

**Authors:** Sung Min Son, So Jung Park, Sophia Y. Breusegem, Delphine Larrieu, David C. Rubinsztein

**Affiliations:** 1 Cambridge Institute for Medical Research, University of Cambridge, Cambridge, UK; 2 UK Dementia Research Institute, Cambridge Institute for Medical Research, University of Cambridge, Cambridge, UK; 3 Department of Pharmacology, University of Cambridge, Cambridge, UK

**Keywords:** Nutrient signalling, Diseases

Correction to: *Nature Cell Biology* 10.1038/s41556-023-01338-y, published online 24 January 2024.

In the version of the article initially published, the top row of images in Fig. 3e (“p300 WT, Complete media”) were duplicates of the images in Extended Data Fig. 1b and have now been replaced with the correct images. In Extended Data Fig. 3b, the bottom row of images (“– Glc → + Glc”) were duplicated from the “+ Comp.C” row in Fig. 3a. The correct images have now been added to Extended Data Fig. 3b. The original Fig. 3 and Extended Data Fig. 3 are available for comparison in the accompanying Supplementary information. These errors have now been corrected in the HTML and PDF versions of the article and do not affect the quantification associated with these figures.

## Supplementary information

Original, uncorrected Fig. 3 and Extended Data Fig. 3

