# Supplementary material for: p300 nucleocytoplasmic shuttling underlies mTORC1 hyperactivation in Hutchinson–Gilford progeria syndrome
Source: Nat Cell Biol. 2024 Jan 24;26(2):235–49. doi: 10.1038/s41556-023-01338-y (PMC10866696; doi:10.1038/s41556-023-01338-y)
Supplement: Supplementary file 1 — Reporting Summary [file 41556_2023_1338_MOESM1_ESM.pdf]

nature portfolio

Corresponding author(s): David C Rubinsztein

Last updated by author(s): Dec 11, 2023

Reporting Summary

Nature Portfolio wishes to improve the reproducibility of the work that we publish. This form provides structure for consistency and transparency in reporting. For further information on Nature Portfolio policies, see our Editorial Policies and the Editorial Policy Checklist.

Statistics

For all statistical analyses, confirm that the following items are present in the figure legend, table legend, main text, or Methods section.

n/a	Confirmed	
☐	☒	The exact sample size (*n*) for each experimental group/condition, given as a discrete number and unit of measurement
☐	☒	A statement on whether measurements were taken from distinct samples or whether the same sample was measured repeatedly
☐	☒	The statistical test(s) used AND whether they are one- or two-sided<br>*Only common tests should be described solely by name; describe more complex techniques in the Methods section.*
☒	☐	A description of all covariates tested
☒	☐	A description of any assumptions or corrections, such as tests of normality and adjustment for multiple comparisons
☐	☒	A full description of the statistical parameters including central tendency (e.g. means) or other basic estimates (e.g. regression coefficient) AND variation (e.g. standard deviation) or associated estimates of uncertainty (e.g. confidence intervals)
☐	☒	For null hypothesis testing, the test statistic (e.g. *F*, *t*, *r*) with confidence intervals, effect sizes, degrees of freedom and *P* value noted<br>*Give P values as exact values whenever suitable.*
☒	☐	For Bayesian analysis, information on the choice of priors and Markov chain Monte Carlo settings
☒	☐	For hierarchical and complex designs, identification of the appropriate level for tests and full reporting of outcomes
☐	☒	Estimates of effect sizes (e.g. Cohen's *d*, Pearson's *r*), indicating how they were calculated

Our web collection on statistics for biologists contains articles on many of the points above.

Software and code

Policy information about availability of computer code

Data collection	Gels were imaged using LICOR-Odyssey apparatus using IMAGE STUDIO Lite Licor ver 5.2, Inc.<br>Confocal Carl Zeiss LSM710, LSM780 and LSM880 operated with ZEN Black imaging software 2.3.
Data analysis	IMAGE STUDIO Lite LI-COR ver 5.2, Inc and Image J (National Institute of Health, USA) for gel analysis.<br>ZEN imaging software (ZEN Black 2.3 Carl Zeiss Microscopy) and Image J ver 1.54f for microscopic image analysis.<br>Volocity 6.3 Software (PerkinElmer) for Mander's Overlap Coefficient (MOC) or Pearson's correlation coefficient (PCC).<br>Microsoft Excel (Excel 2016 Microsoft office) and GraphPad Prism v7 (GraphPad Software) for statistical analysis.

For manuscripts utilizing custom algorithms or software that are central to the research but not yet described in published literature, software must be made available to editors and reviewers. We strongly encourage code deposition in a community repository (e.g. GitHub). See the Nature Portfolio guidelines for submitting code & software for further information.

Data

Policy information about availability of data

All manuscripts must include a data availability statement. This statement should provide the following information, where applicable:

- Accession codes, unique identifiers, or web links for publicly available datasets
- A description of any restrictions on data availability
- For clinical datasets or third party data, please ensure that the statement adheres to our policy

All data supporting the findings of this study are available from the corresponding author upon reasonable request. Source data are provided with this paper.

Research involving human participants, their data, or biological material

Policy information about studies with human participants or human data. See also policy information about sex, gender (identity/presentation), and sexual orientation and race, ethnicity and racism.

Reporting on sex and gender	not applicable
Reporting on race, ethnicity, or other socially relevant groupings	not applicable
Population characteristics	not applicable
Recruitment	not applicable
Ethics oversight	not applicable

Note that full information on the approval of the study protocol must also be provided in the manuscript.

Field-specific reporting

Please select the one below that is the best fit for your research. If you are not sure, read the appropriate sections before making your selection.

☒ Life sciences ☐ Behavioural & social sciences ☐ Ecological, evolutionary & environmental sciences

For a reference copy of the document with all sections, see nature.com/documents/nr-reporting-summary-flat.pdf

Life sciences study design

All studies must disclose on these points even when the disclosure is negative.

Sample size	Sample sizes were chosen on the basis of extensive experience with the assays we have performed. (e.g. Son et al. Cell Metabolism 2019; Son et al. Nature Communications 2020; Wrobel et al. Nature Communications 2022)
Data exclusions	No data were excluded from the analysis.
Replication	All experiments were repeated by at least three times, and all experiments were reproducible. We used the average of each triplicate as a biological replicate for statistical analyses.
Randomization	All in vivo experiments and tests were randomly assigned, but no randomization was performed for cell culture experiments.
Blinding	Immunofluorescence analysis was blinded when possible. Western blot analysis was not blinded as it was not possible as the gel loading order needs to be defined. Investigators were not blinded during the other experiments.

Reporting for specific materials, systems and methods

We require information from authors about some types of materials, experimental systems and methods used in many studies. Here, indicate whether each material, system or method listed is relevant to your study. If you are not sure if a list item applies to your research, read the appropriate section before selecting a response.

Materials & experimental systems

n/a	Involved in the study
☐	☒ Antibodies
☐	☒ Eukaryotic cell lines
☒	☐ Palaeontology and archaeology
☐	☒ Animals and other organisms
☒	☐ Clinical data
☒	☐ Dual use research of concern
☒	☐ Plants

Methods

n/a	Involved in the study
☒	☐ ChIP-seq
☒	☐ Flow cytometry
☒	☐ MRI-based neuroimaging

Antibodies

Antibodies used

Primary antibodies: mouse anti-Flag M2 (#F3165, RRID:AB_262044, WB 1:2000), rabbit anti-Actin (#A2066, RRID AB_476693, WB 1:2000), rabbit anti-HA tag (#AP1012A, RRID:AB_352510, WB 1:2000), mouse anti-progerin (#05-1231, RRID:AB_1587236, WB 1:1000) and mouse anti-α-Tubulin (#T9026, RRID:AB_477593, WB 1:3000) from Sigma Aldrich; mouse anti-GAPDH clone 6C5 (#ab8245, RRID:AB_2107448, WB 1:3000), rabbit anti-p300 (#ab10485, RRID:AB_297224, WB 1:1000, IF 1:200), rabbit anti-phospho-p300 (Ser89; #ab135554, DB 1:500, IF 1:100), rabbit anti-CBP (#ab137334, RRID:AB_2916306, WB 1:1000), rabbit anti-GFP tag (#ab6556, RRID:AB_305564, WB 1:2000, IF 1:200), rabbit anti-H2B (acK16; #ab177427; WB 1:1000), rabbit anti-H4 (#ab7311,RRID:AB_305837, WB 1:1000), rat anti-LAMP1 (#ab25630, RRID:AB_470708, IF 1:300), rabbit anti-LAMP1 (#ab24170, RRID:AB_775978, IF 1:400), rabbit anti-acetylated-Lysine (Ac-K) (#ab21623, RRID:AB_446436, WB 1:500), mouse anti-6X His tag (#ab18184, RRID:AB_444306, WB 1:1000), rabbit anti-Lamin B1 (#ab16048, RRID:AB_443298, WB 1:1000), mouse anti-Myc tag (#ab32, RRID:AB_303599, WB 1:1000), rabbit anti-14-3-3zeta (#ab51129, RRID:AB_867447, WB 1:1000), rabbit anti-Histone H3 (tri-methyl K9; #ab8898, RRID:AB_306848, WB 1:1000, IF 1:200), mouse anti-BAT3 (#ab88292, RRID:AB_2040593, IF 1:300), rabbit anti-Vps34 (# ab227861, RRID: AB_2827796; WB 1:1000), rabbit anti-ATG7 (Cat# ab133528, RRID:AB_2532126; WB 1:1000), rabbit anti-LC3B (#ab51520, RRID:AB_881429; IF 1:400) and rabbit anti-ALX1 (#ab181101, IF 1:300) from Abcam; goat anti-HA (#NB600-362, RRID:AB_10124937, 1:1000) and rabbit anti-LC3B (#NB100-2220, RRID: AB_10003146, WB 1:1000) from Novus Biologicals; rabbit anti-p300 (#sc-585, RRID:AB_2231120, #sc-48343, RRID:AB_628075, IF 1:50), goat anti-Lamin B (#sc-6217, RRID:AB_648158, WB 1:1000), rabbit anti-p-PP2Ac (#sc-271903, RRID:AB_10611810, WB 1:1000), mouse anti-PP1γ (#sc-515943, RRID:AB_2909495, WB 1:1000), mouse anti-CRM1 (#sc-74454, RRID:AB_1122704, WB 1:500, IF 1:100), mouse anti-TOM20 (#sc-17764, RRID:AB_628381, IF 1:100) from SantaCruz Biotechnology; mouse anti-GFP (#632375 and #632592, RRID:AB_2756343, IF 1:100) from Clontech; mouse anti-HA.11 clone 16B12 (#MMS-101P, RRID:AB_10064068, WB 1:1000) from Covance; mouse anti-p300 (#05-257, RRID:AB_309670, WB 1:1000, IF 1:100) from Millipore; mouse anti- acetylated-Lysine (Ac-K) (#AAC01, RRID:AB_2884959, WB 1:1000) from Cytoskeleton, Inc; mouse anti-GFP tag (#66002-1-Ig, RRID:AB_11182611, WB 1:1000, IF 1:100), rabbit anti-Lamin A/C (#10298-1-AP, RRID:AB_2296961, IF 1:100), rabbit anti-Lamin B1 (#12987-1-AP, RRID:AB_2136290, IF 1:100) and mouse anti-mTOR (#66888-1-Ig, RRID:AB_2882219, IF 1:400) from Proteintech; rabbit anti-p300 (#86377, RRID:AB_2800077, IF 1:300), rabbit anti-CBP (#7389, RRID:AB_2616020, WB 1:1000), rabbit anti-acetyl-CBP (Lys1535)/p300 (Lys1499) (#4771, RRID:AB_2262406, WB 1:1000), rabbit anti-GCN5 (#3305, RRID:AB_2128281, 1:1000), rabbit anti-PCAF (#3378, RRID:AB_2128409, WB 1:1000), rabbit anti-Histone H2B (#12364, RRID:AB_2714167, WB 1:1000), rabbit anti-Histone H3 (#9715, RRID:AB_331563, WB 1:1000), rabbit anti-H3 (acK9; #9649, RRID:AB_823528, WB 1:1000), rabbit anti-H3 (acK56; #4243, RRID:AB_10548193, WB 1:1000), rabbit anti-H4 (acK12; #2591, RRID:AB_2118617, WB 1:1000), rabbit anti-H4 (acK16; #13534, RRID:AB_2687581, WB 1:1000), rabbit anti-LAMP1 (#9091, RRID:AB_2687579, WB 1:1000, IF 1:200), rabbit anti-mTOR (#2972, RRID:AB_330978, WB 1:1000; #2983, RRID:AB_2105622, IF 1:400), rabbit anti-raptor (#2280, RRID:AB_561245, WB 1:1000, IF 1:100), rabbit anti-phospho-S6K1 (Thr389; #9234, RRID:AB_2269803, WB 1:1000), anti-total S6K1 (#9202, RRID:AB_331676, WB 1:1000), rabbit anti-phospho-S6 Ribosomal Protein (p-S6) (Ser235/236; #4856, RRID:AB_2181037, WB 1:1000, IF 1:200), rabbit anti-S6 Ribosomal Protein (S6) (#2217, RRID:AB_331355, WB 1:1000), rabbit anti-phospho-4E-BP1 (Thr37/46; #9459, RRID:AB_330985, WB 1:1000), rabbit anti-4E-BP1 (#9452, RRID:AB_331692, WB 1:1000), rabbit anti-acetylated-Lysine (Ac-K) (#9814, RRID:AB_10544700; #9441, RRID:AB_331805, WB 1:1000), rabbit anti-PP2Ac (#2259, RRID:AB_561239, WB 1:1000), rabbit anti-phospho-ACC1 (Ser79; #11818, RRID:AB_2687505, WB 1:1000), rabbit anti-ACC (#3676, RRID:AB_2219397, WB 1:1000), rabbit anti-phospho-AMPK (Thr172; #2531, RRID:AB_330330, 1:1000), rabbit anti-AMPK (#2532, RRID:AB_330331, WB 1:1000), rabbit anti-phospho-Histone H2A.X (Ser139; #2577, RRID:AB_2118010, WB 1:1000; #9718, RRID:AB_2118009, IF 1:200) from Cell Signaling Technology.
Secondary Antibodies: anti-mouse (#NA931V, RRID:AB_772210) and anti-rabbit (#NA934V) horseradish peroxidise (HRP)-conjugated secondary antibodies (GE Healthcare); anti-goat horseradish peroxidise (HRP)-conjugated secondary antibody (#611620, RRID:AB_87867, Invitrogen/Life Technologies). For immunoflourescence, goat-anti-mouse Alexa Fluor 488 (#A11029, RRID:AB_2534088, 1:400), 555 (#A21147, RRID:AB_1500897, 1:400) and 594 (#A11032, RRID:AB_2534091, 1:400), goat-anti-rabbit Alexa Fluor 488 (#A32731, RRID:AB_2633280, 1:400) and 555 (#A21428, RRID:AB_141784, 1:400) from ThermoFisher Scientific.

Validation

All antibodies used in this study were purchased from commercial vendors who had validated specificity in human cells/ mouse tissues for the specific assays (Western blot, immunoprecipitation and/or immunofluorescence). It is described on data sheets and online.
We have confirmed that the LC3 antibody does not detect an LC3-II band in autophagy null cells.

Eukaryotic cell lines

Policy information about cell lines and Sex and Gender in Research

Cell line source(s)

Human cervical epithelium HeLa (ATCC; #CCL-2; CVCL_0030), human neuroblastoma SH-SY5Y (ECACC; #94030304), human embryonic kidney cell line HEK293 (ECACC; #85120602), Human retinal pigment epithelium RPE1 (ATCC, #CRL-4000), human breast cancer cell line MCF7 (ATCC; #HTB-22), human lung carcinoma A549 cells (kindly provided by Dr. F. Buss (University of Cambridge, UK)), human neuroglioma H4 cells (ATCC; #HTB-148), Human hepatoma HepG2 cells (ECACC; #85011430), and

	Human mammary epithelium MCF10A cells were purchased from Horizon (#HD PAR-058).<br>AMPK α1/α2 double knockout (dKO) MEFs were a gift from B. Viollet (Universite´ Paris Descartes).<br>Primary mouse embryonic fibroblasts (MEFs) were isolated from wild-type C57BL/6 mouce embryos at E12.<br>Unaffected fibroblast control (Coriell Institute #GM05565, #GM02036, #GM00969)<br>Primary human dermal fibroblasts from patients with HGPS (1) (Coriell Institute #AG01972)<br>Primary human dermal fibroblasts from patients with HGPS (2) (Coriell Institute #AG11513)
Authentication	The cell lines were ordered from ATCC, Horizon or Coriell Institute with authentication.<br>HeLa authenticatd by ATCC (by Short Tandem Repeat (STR) profiling; FTA barcode:STRA1466)<br>HEK293 authenticatd by LGC (STR profiling, FTA barcode:STRA1472)<br>SH-SY5Y authenticatd by LGC (STR profiling, FTA barcode:STRA1440)<br>RPE1 authenticatd by ATCC (STR profiling)<br>MCF7 authenticatd by ATCC (STR profiling)<br>H4 authenticatd by ATCC (STR profiling)<br>HepG2 authenticatd by ECACC (STR profiling)<br>MCF10A authenticatd by Horizon (STR profiling)<br>AMPK dKO MEF were authenticatd by Western blot analysis with AMPK antibody.<br>Primary MEFs were not authenticated by STR.
Mycoplasma contamination	All the cells were regularly tested using EZ-PCR Mycoplasma Test Kit (Biological Industries; cat#20-700-20). Cells used in this study were mycoplasma negative.
Commonly misidentified lines<br>(See ICLAC register)	no commonly misidentified cell lines were used in the study.

Animals and other research organisms

Policy information about studies involving animals; ARRIVE guidelines recommended for reporting animal research, and Sex and Gender in Research

Laboratory animals	Wild type C57Bl/6J mice (6 to 7-weeks-old)<br>Mice were housed in individually ventilated cages with free access to standard animal food chow (#R105; SAFE) and water, in a climate-controlled room with a 12 h light/dark cycle, except when subjected to starvation-refeeding protocols.
Wild animals	No wild animals were used in the study.
Reporting on sex	The ratio of sexes of used mice was 1:1 and the number of the mice used for the experiments are indicated for each experiment in the figure legends (in general, n=6).
Field-collected samples	No field collected samples were used in the study.
Ethics oversight	Mouse studies and procedures were performed in accordance with the UK Animals (Scientific Procedures) Act with appropriate Home Office Project and Personal animal licenses and with the approval of the University of Cambridge Animal welfare and Ethical Review Body.

Note that full information on the approval of the study protocol must also be provided in the manuscript.
