# Supplementary figures and images for: p300 nucleocytoplasmic shuttling underlies mTORC1 hyperactivation in Hutchinson–Gilford progeria syndrome
Source: Nat Cell Biol. 2024 Jan 24;26(2):235–49. doi: 10.1038/s41556-023-01338-y (PMC10866696; doi:10.1038/s41556-023-01338-y)

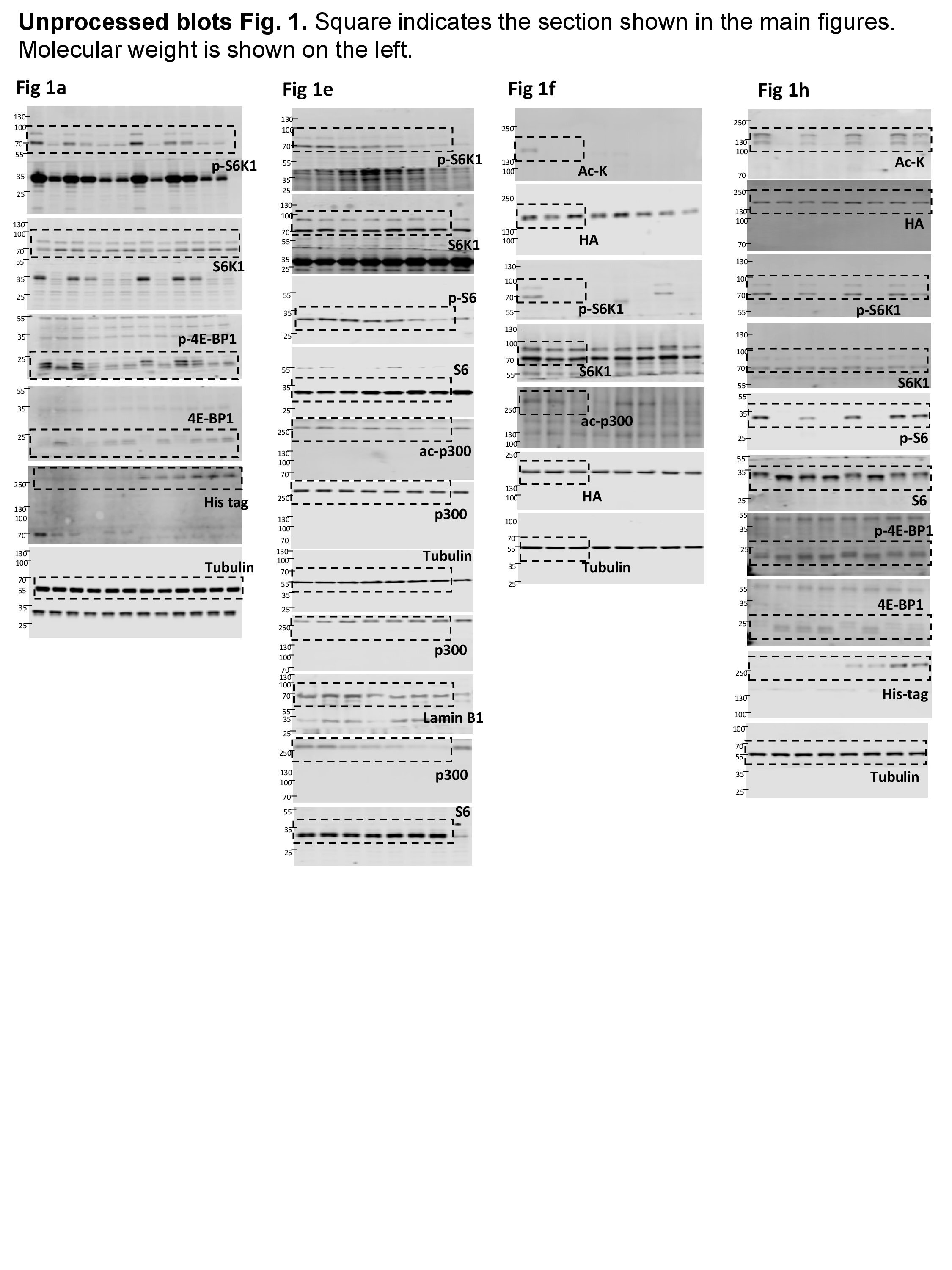

Supplement: Supplementary file 4 — Uncropped western gels for Fig. 1. [file 41556_2023_1338_MOESM4_ESM.jpg]

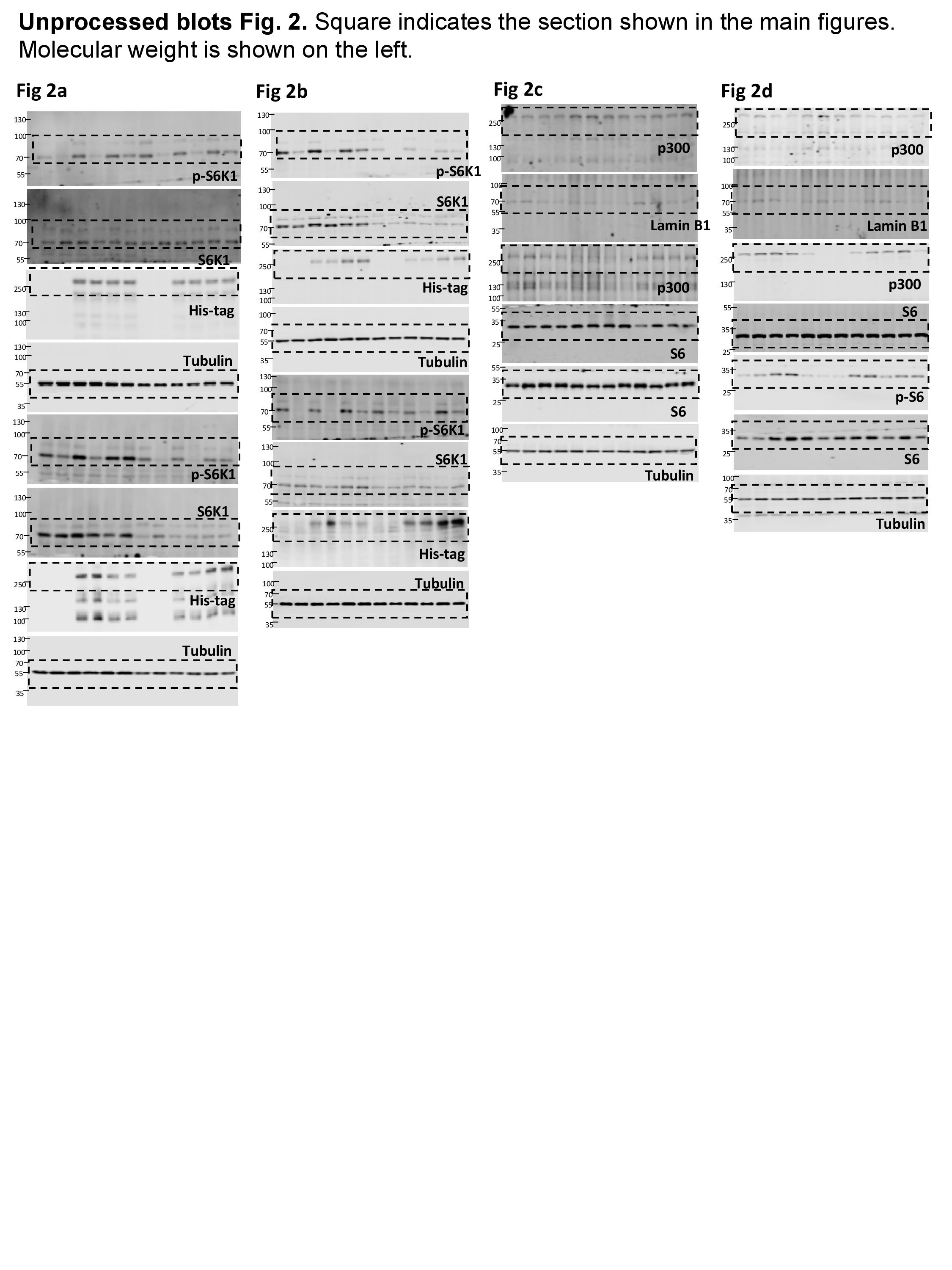

Supplement: Supplementary file 5 — Uncropped western gels for Fig. 2. [file 41556_2023_1338_MOESM5_ESM.jpg]

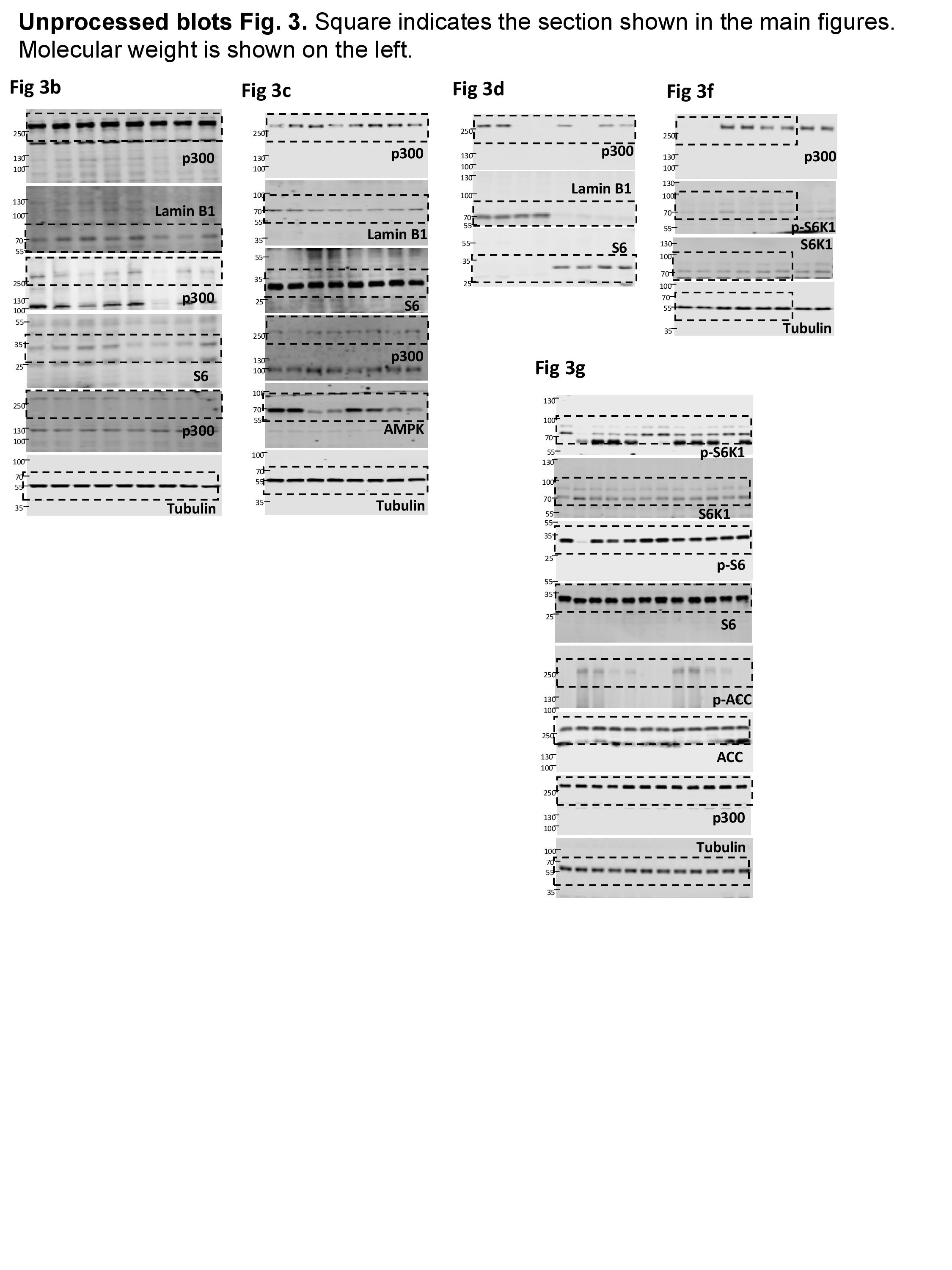

Supplement: Supplementary file 6 — Uncropped western gels for Fig. 3. [file 41556_2023_1338_MOESM6_ESM.jpg]

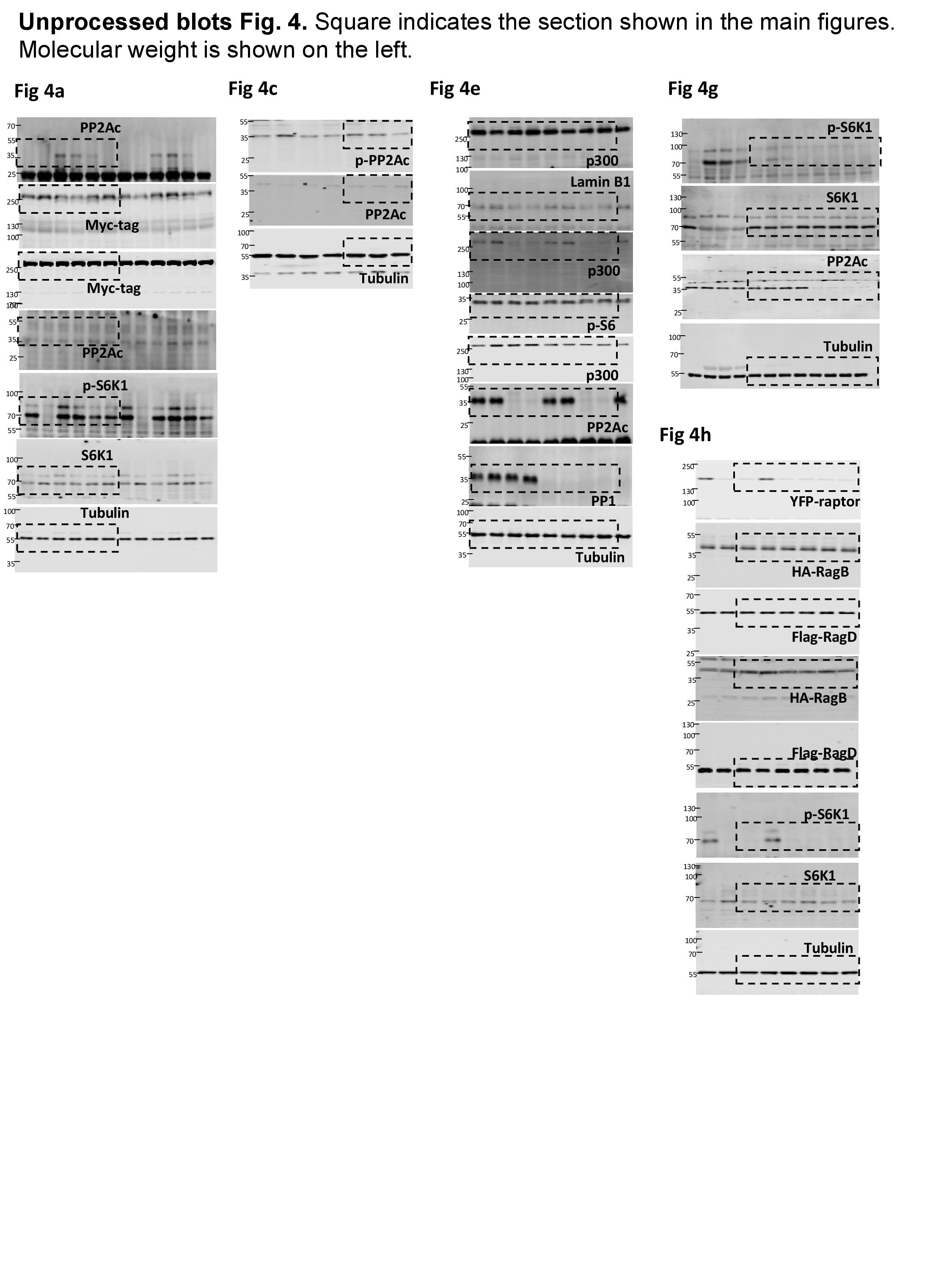

Supplement: Supplementary file 7 — Uncropped western gels for Fig. 4. [file 41556_2023_1338_MOESM7_ESM.jpg]

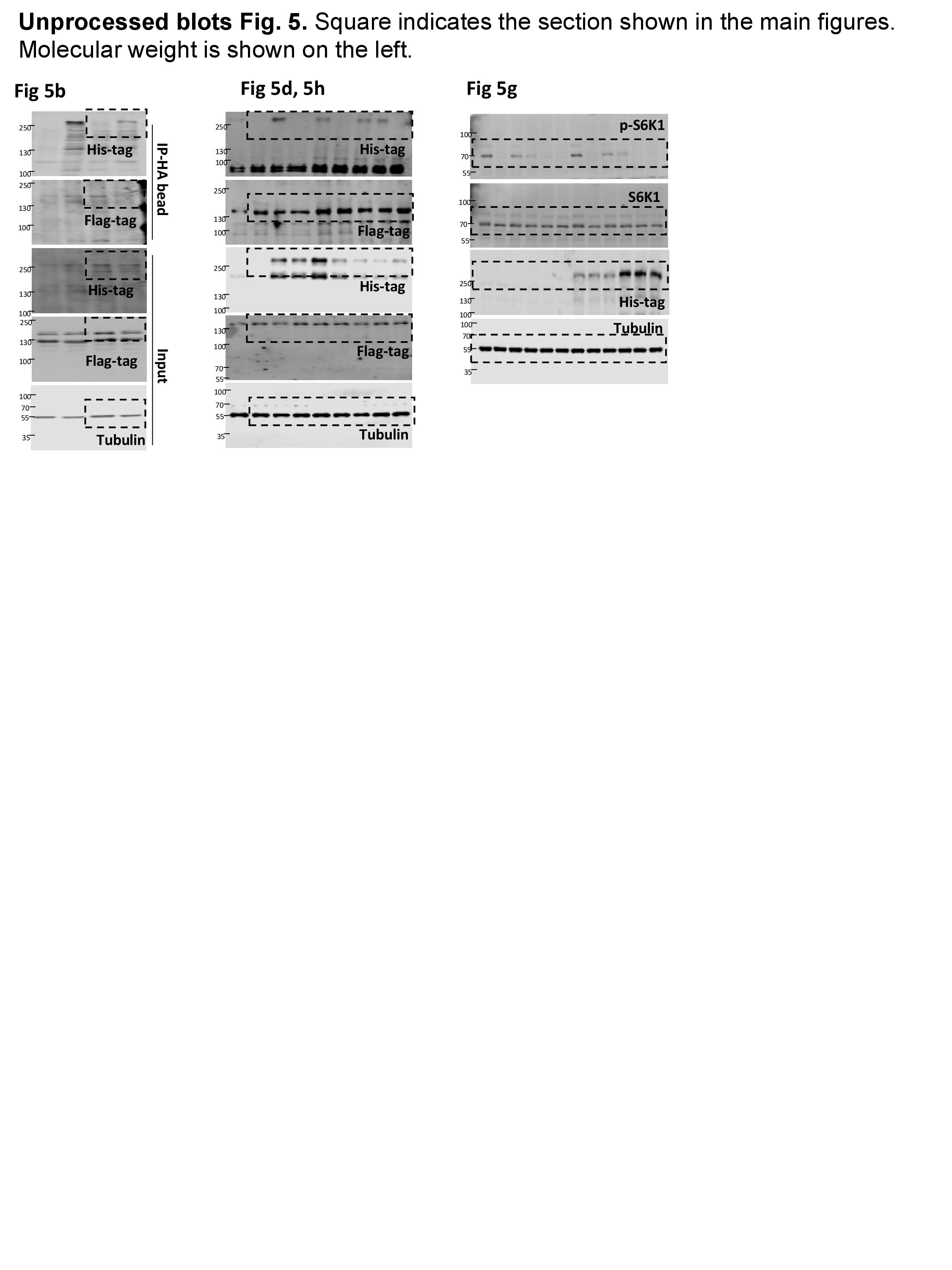

Supplement: Supplementary file 8 — Uncropped western gels for Fig. 5. [file 41556_2023_1338_MOESM8_ESM.jpg]

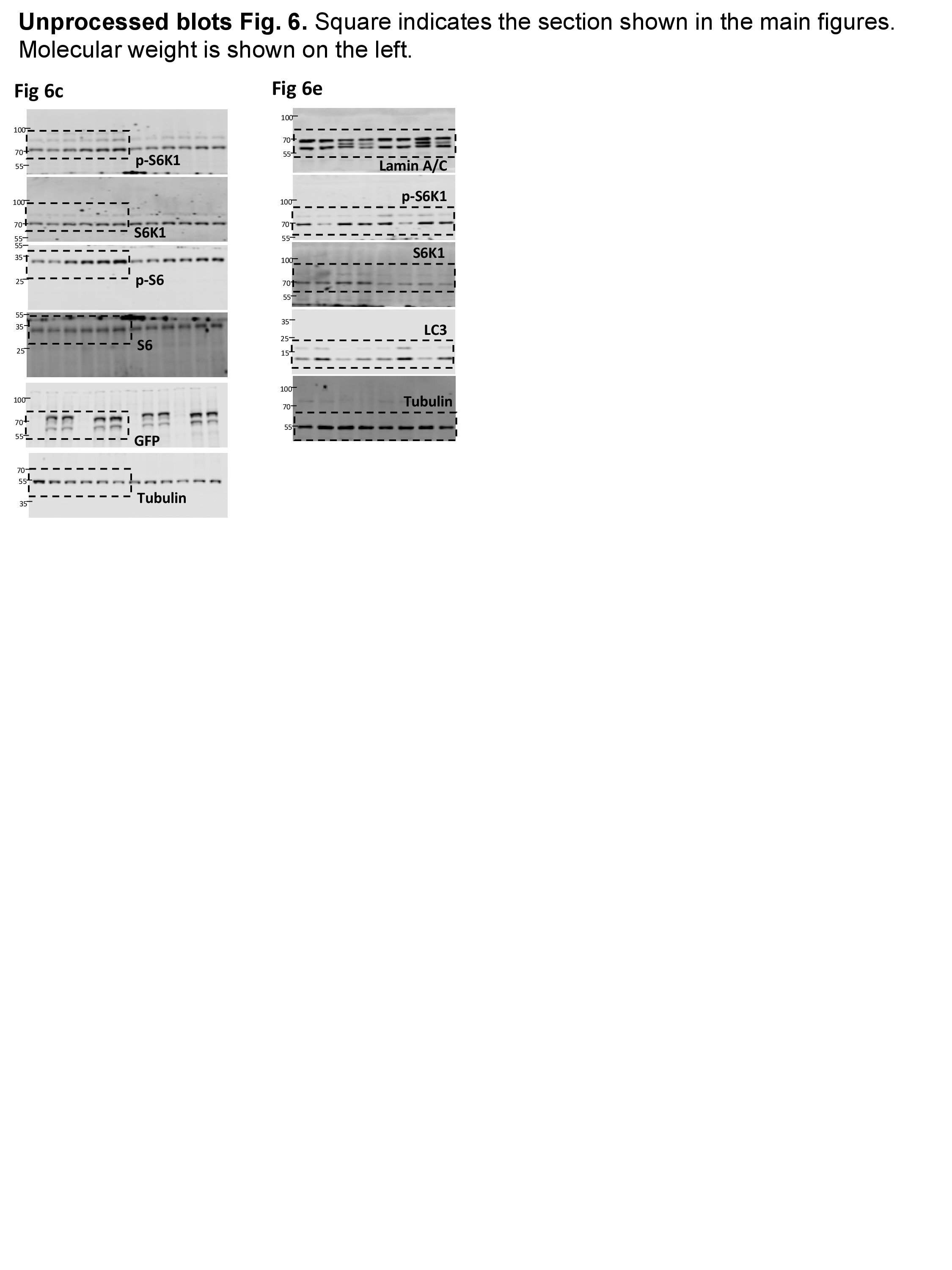

Supplement: Supplementary file 9 — Uncropped western gels for Fig. 6. [file 41556_2023_1338_MOESM9_ESM.jpg]

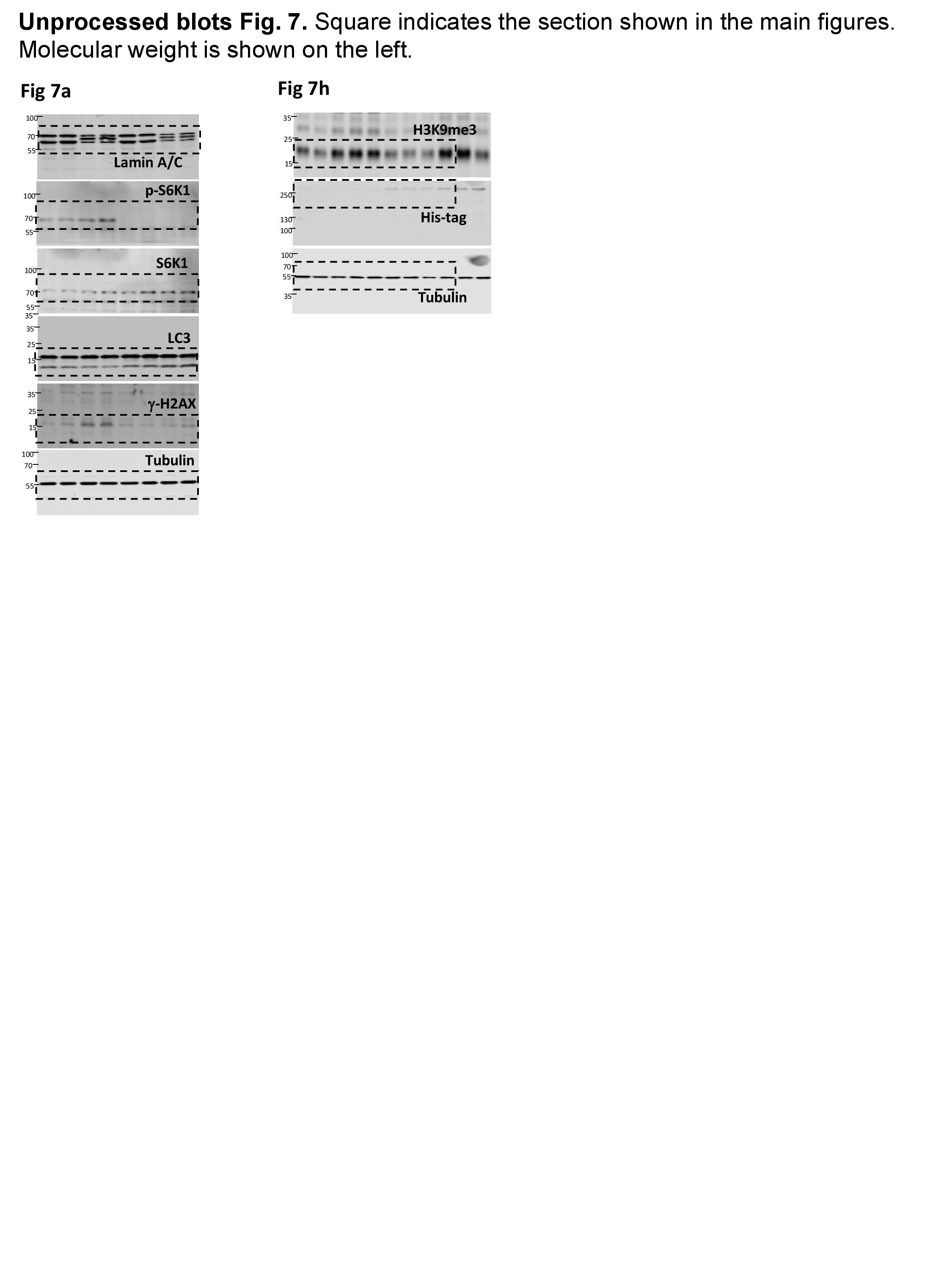

Supplement: Supplementary file 10 — Uncropped western gels for Fig. 7. [file 41556_2023_1338_MOESM10_ESM.jpg]

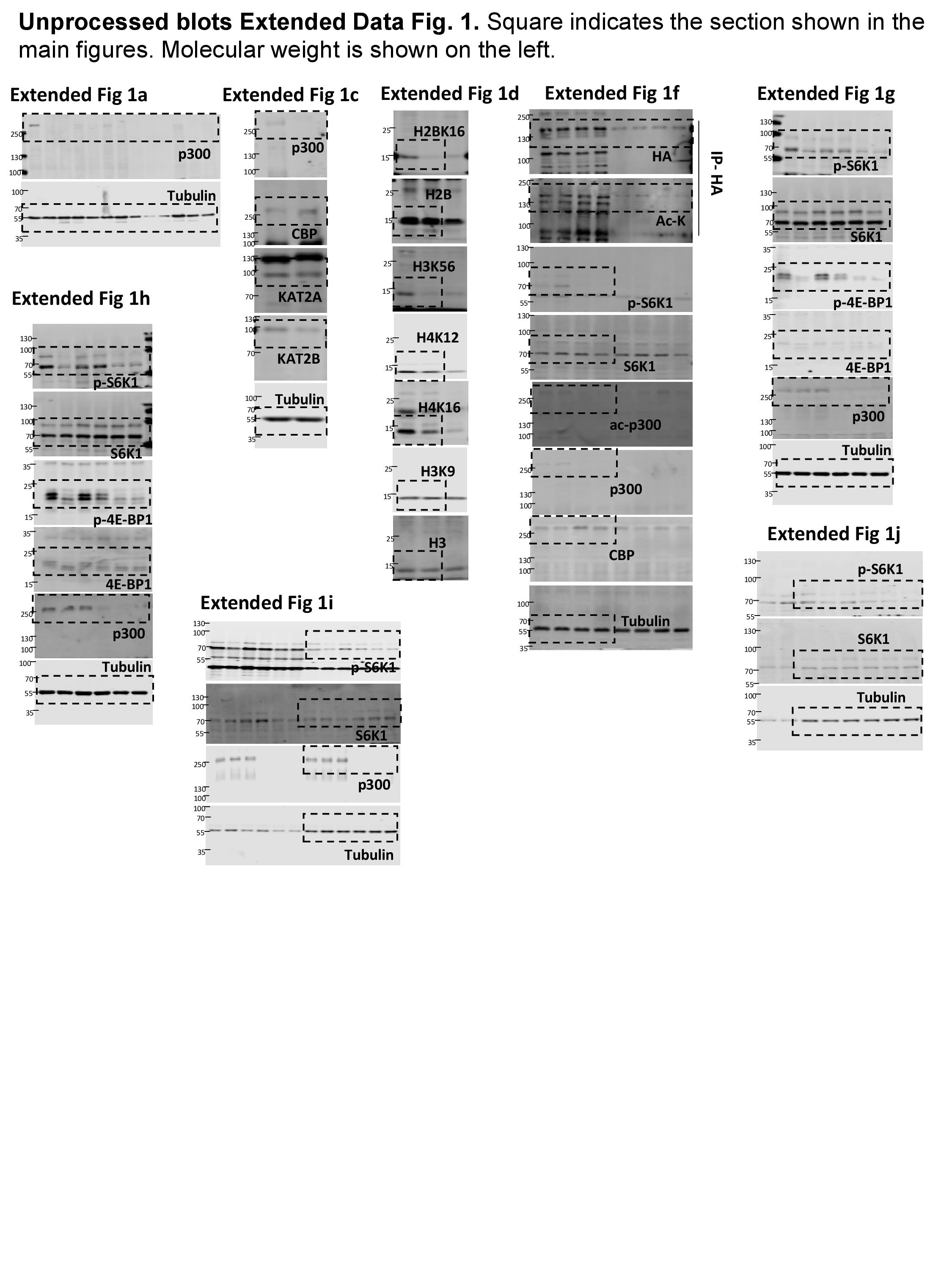

Supplement: Supplementary file 11 — Uncropped western gels for ED1. [file 41556_2023_1338_MOESM11_ESM.jpg]

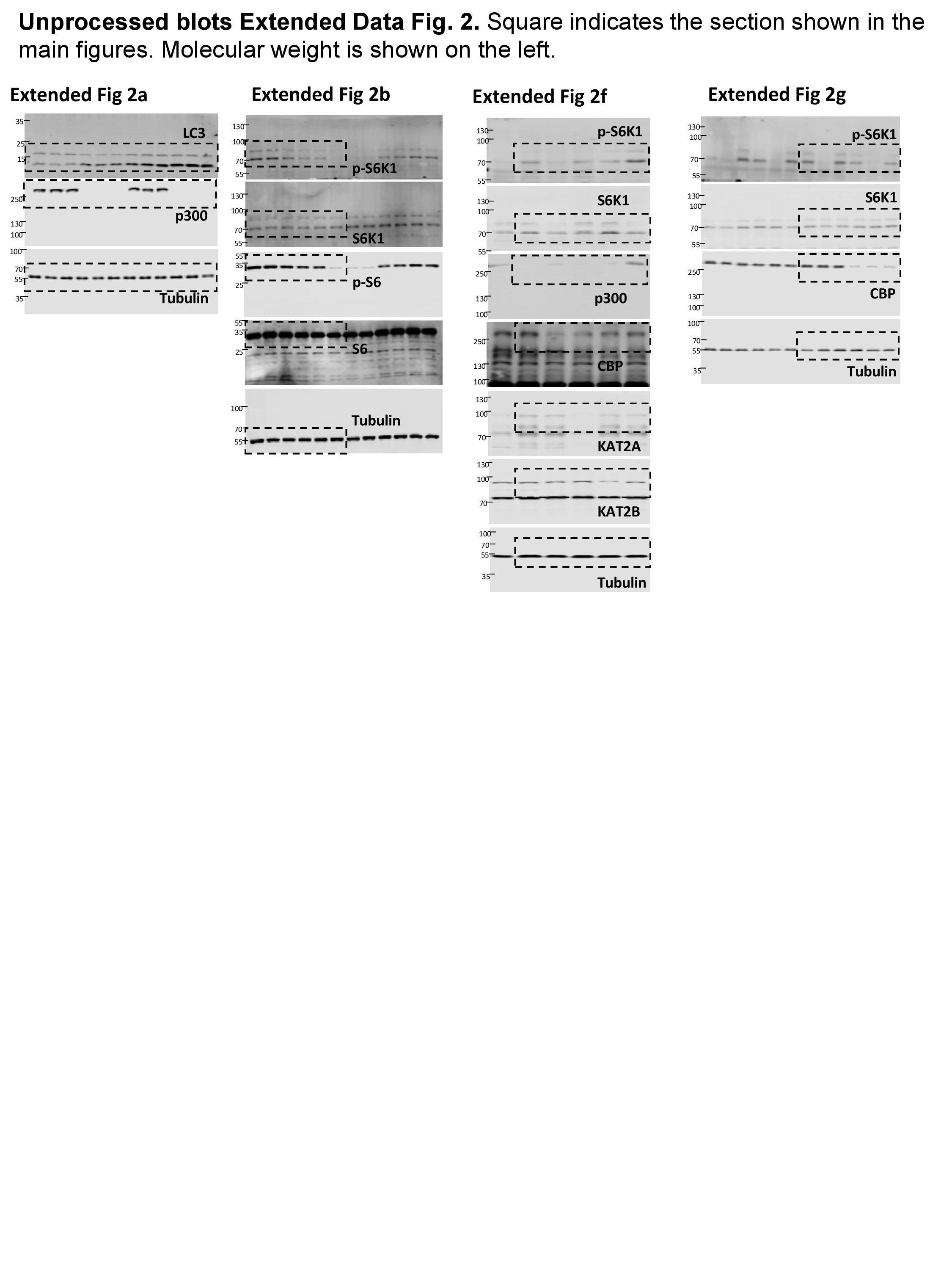

Supplement: Supplementary file 12 — Uncropped western gels for ED1. [file 41556_2023_1338_MOESM12_ESM.jpg]

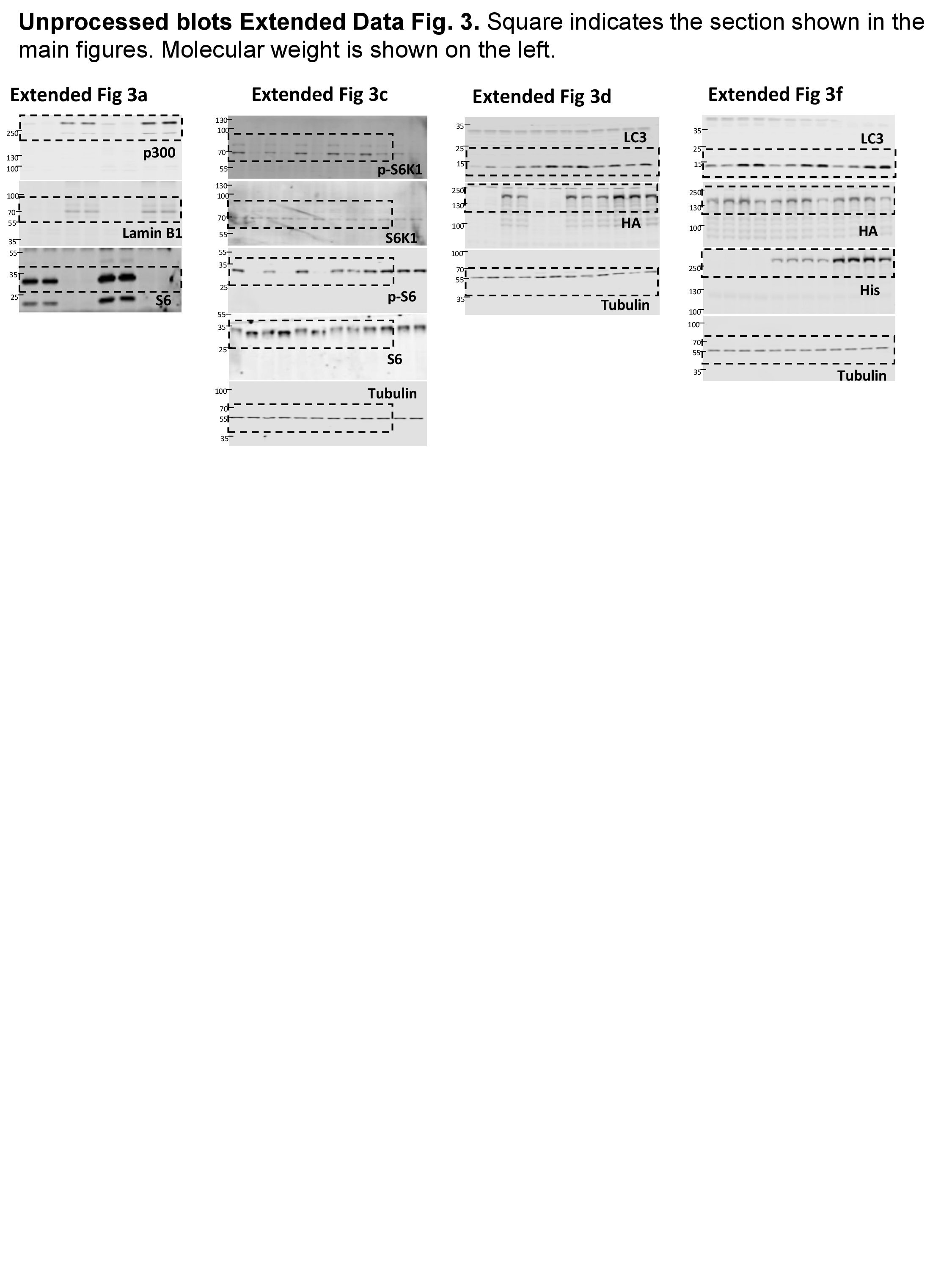

Supplement: Supplementary file 13 — Uncropped western gels for ED1. [file 41556_2023_1338_MOESM13_ESM.jpg]

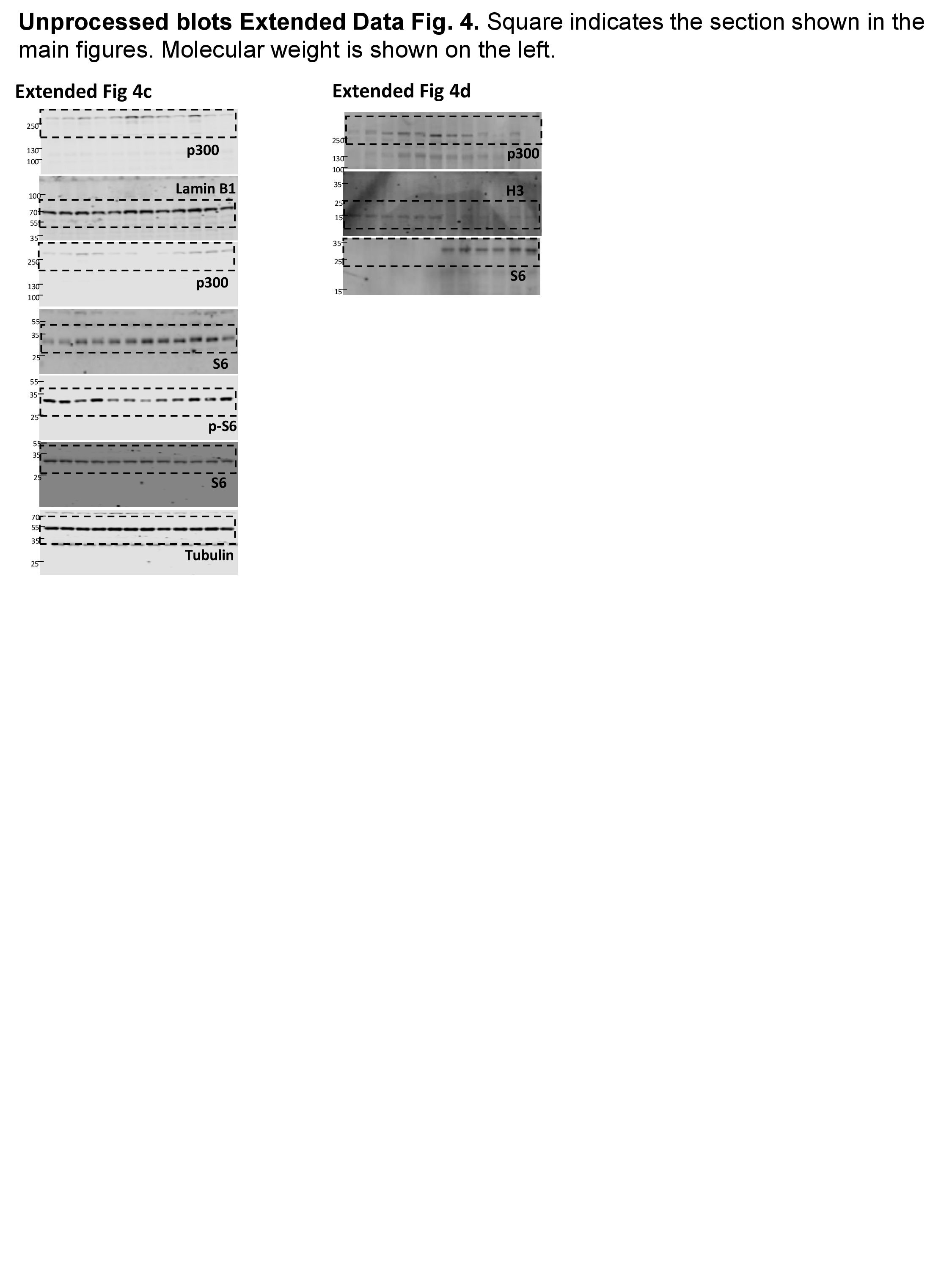

Supplement: Supplementary file 14 — Uncropped western gels for ED1. [file 41556_2023_1338_MOESM14_ESM.jpg]

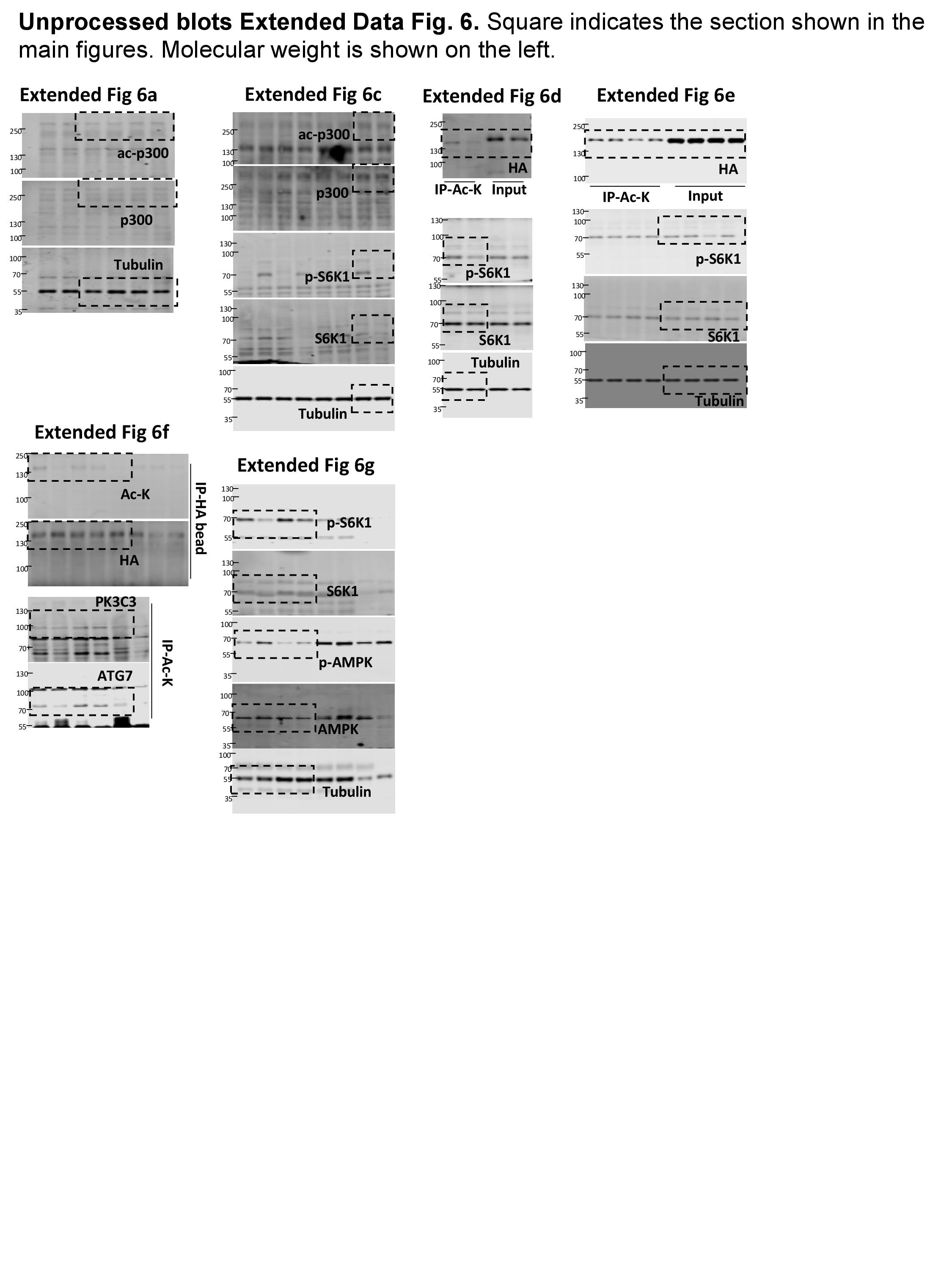

Supplement: Supplementary file 15 — Uncropped western gels for ED1. [file 41556_2023_1338_MOESM15_ESM.jpg]

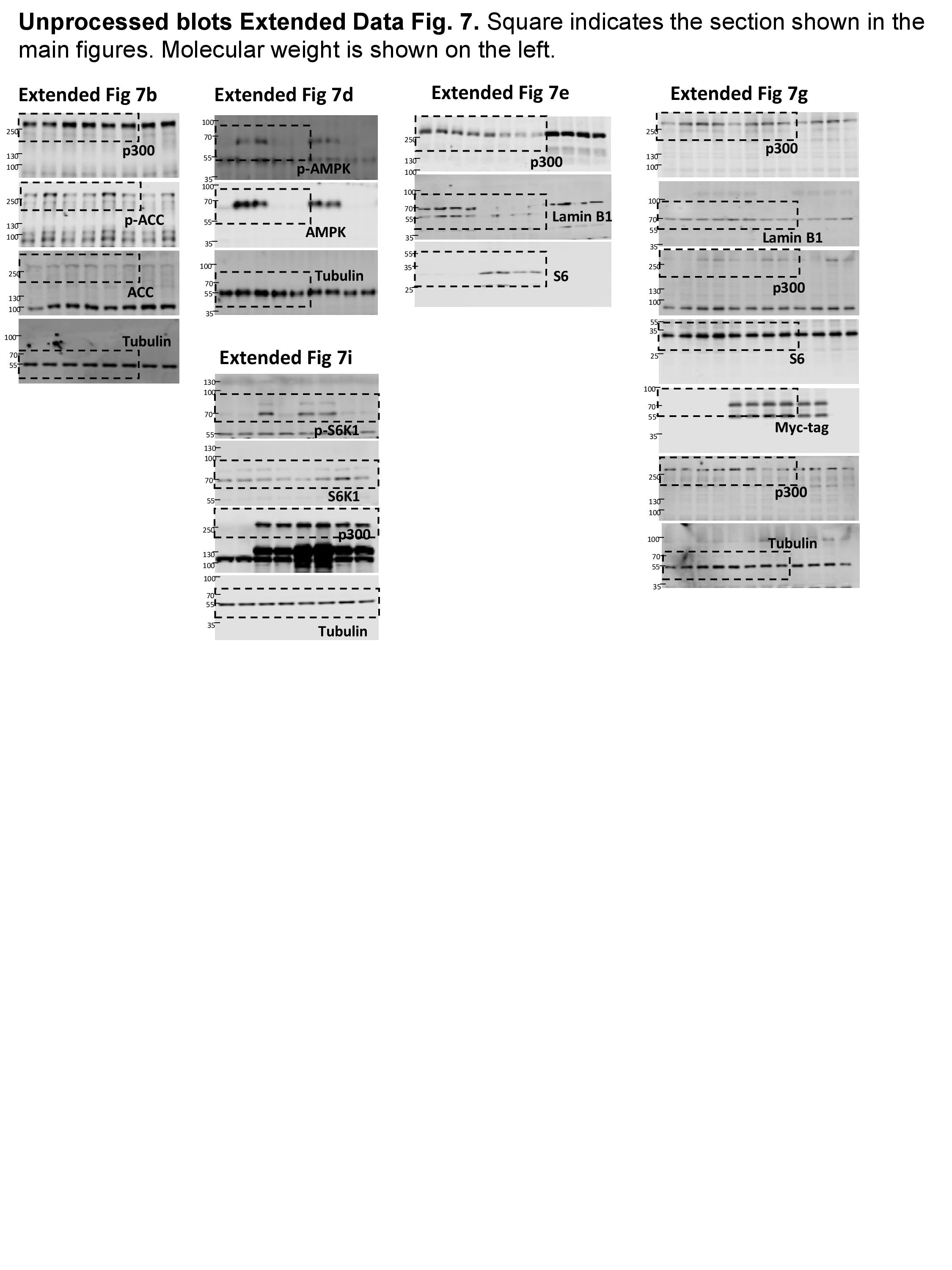

Supplement: Supplementary file 16 — Uncropped western gels for ED1. [file 41556_2023_1338_MOESM16_ESM.jpg]

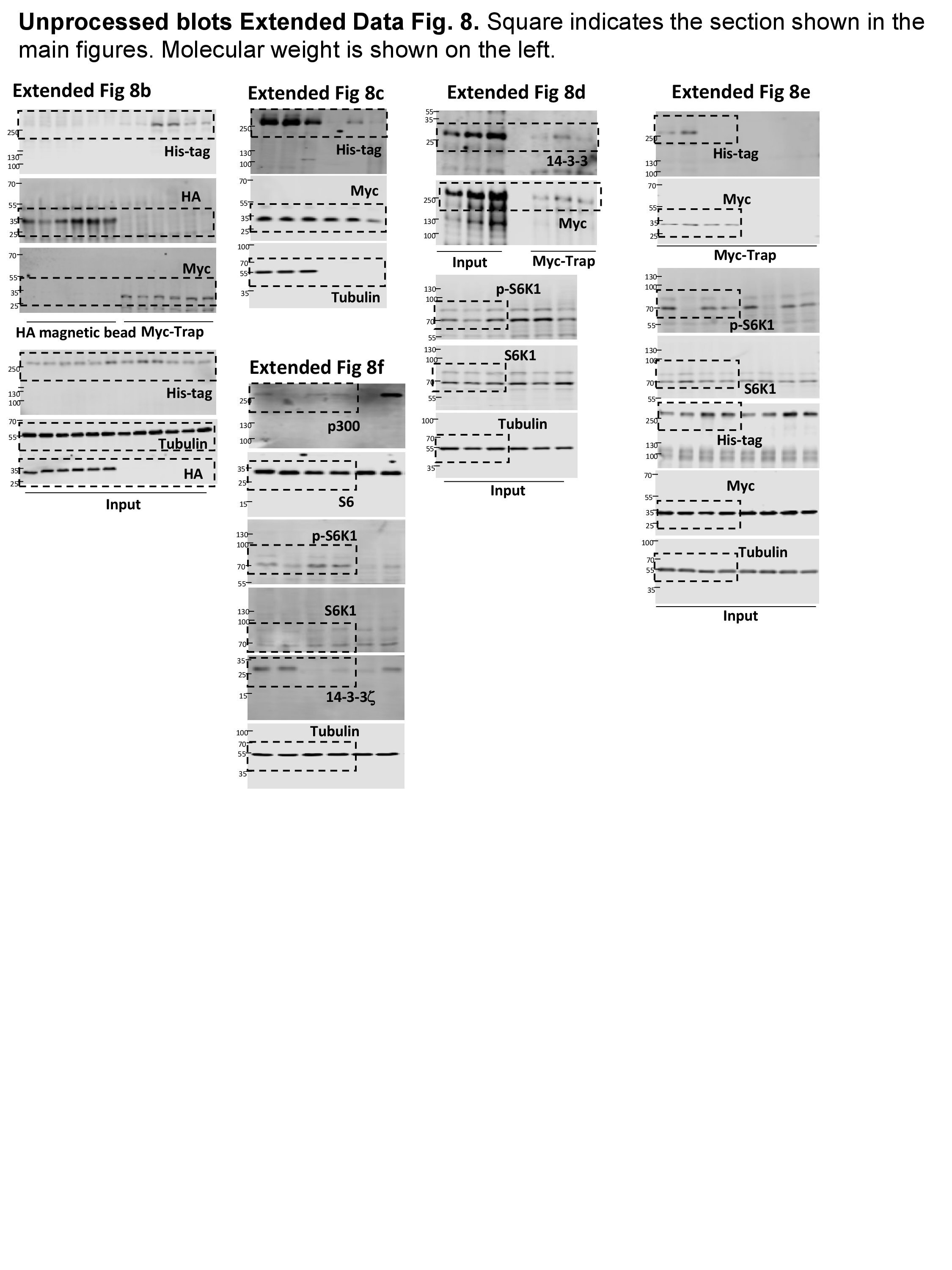

Supplement: Supplementary file 17 — Uncropped western gels for ED1. [file 41556_2023_1338_MOESM17_ESM.jpg]

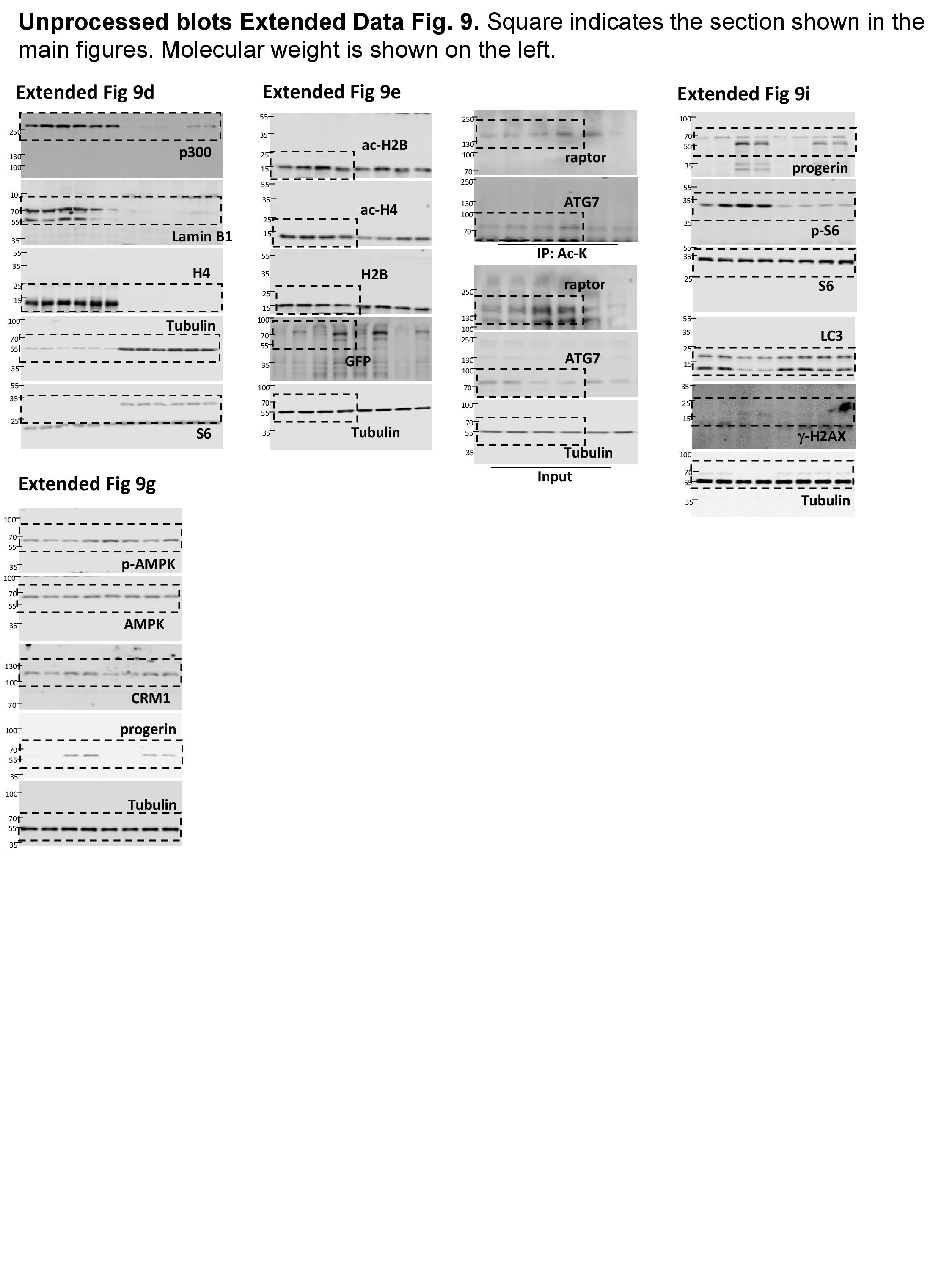

Supplement: Supplementary file 18 — Uncropped western gels for ED1. [file 41556_2023_1338_MOESM18_ESM.jpg]

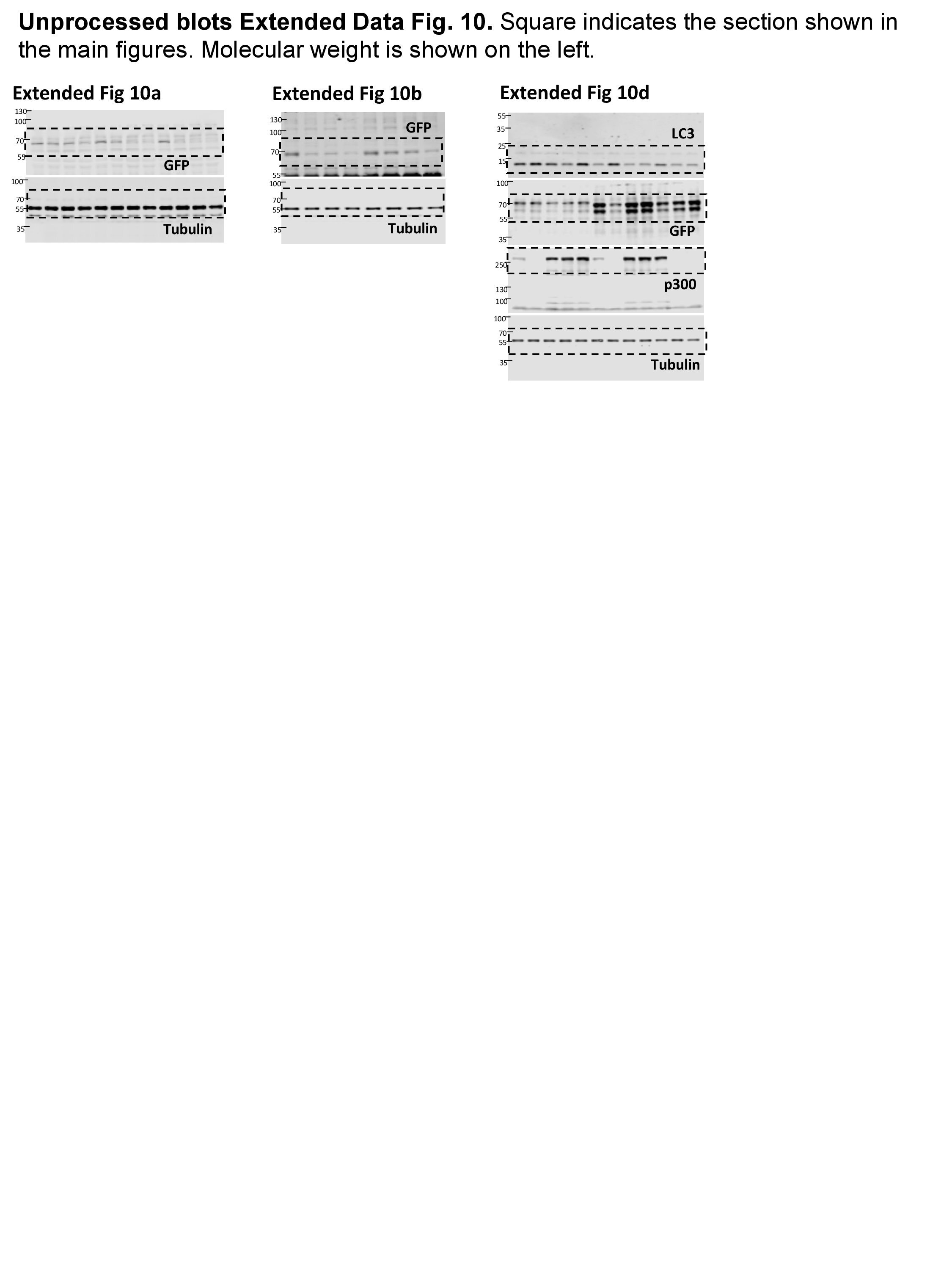

Supplement: Supplementary file 19 — Uncropped western gels for ED1. [file 41556_2023_1338_MOESM19_ESM.jpg]
