# Supplementary material for: Author Correction: p300 nucleocytoplasmic shuttling underlies mTORC1 hyperactivation in Hutchinson–Gilford progeria syndrome
Source: Nat Cell Biol. 2025 Nov 28;28(1):207. doi: 10.1038/s41556-025-01850-3 (PMC12807851; doi:10.1038/s41556-025-01850-3)
Supplement: Supplementary file 1 — Original, uncorrected Fig. 3 and Extended Data Fig. 3 [file 41556_2025_1850_MOESM1_ESM.pdf]

nature cell biology

Supplementary information

Author Correction: p300 nucleocytoplasmic shuttling underlies mTORC1 hyperactivation in Hutchinson–Gilford progeria syndrome

In the format provided by the authors and unedited

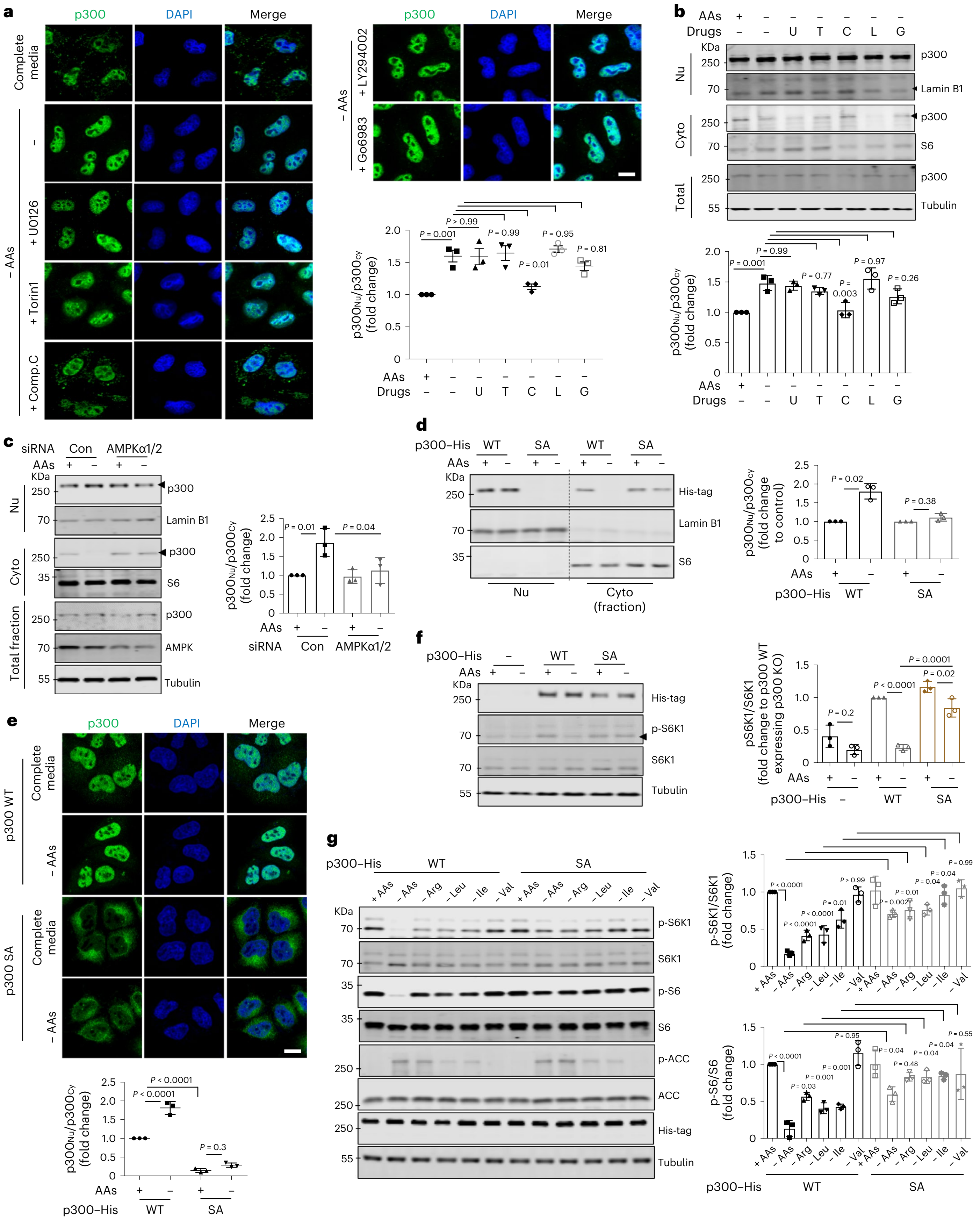
a
p300
DAPI
Merge
Complete media
– AAs
–
+ U0126
+ Torin1
+ Comp.C
+ LY294002
+ Go6983
P = 0.001
P > 0.99
P = 0.99
P = 0.01
P = 0.95
P = 0.81
p300Nu/p300Cy (fold change)
AAs
Drugs
U
T
C
L
G
b
KDa
Nu
Cyto
Total
p300
Lamin B1
S6
Tubulin
P = 0.001
P = 0.99
P = 0.77
P = 0.003
P = 0.97
P = 0.26
c
siRNA
Con
AMPKα1/2
Total fraction
AMPK
P = 0.01
P = 0.04
d
p300–His
WT
SA
His-tag
Nu
Cyto (fraction)
P = 0.02
P = 0.38
p300Nu/p300Cy (fold change to control)
e
p300 WT
p300 SA
P < 0.0001
P = 0.3
f
p-S6K1
S6K1
P = 0.2
P < 0.0001
P = 0.0001
P = 0.02
pS6K1/S6K1 (fold change to p300 WT expressing p300 KO)
g
+ AAs
– AAs
– Arg
– Leu
– Ile
– Val
p-S6
p-ACC
ACC
p-S6K1/S6K1 (fold change)
p-S6/S6 (fold change)
P > 0.99
P = 0.95
P = 0.55

Fig. 3

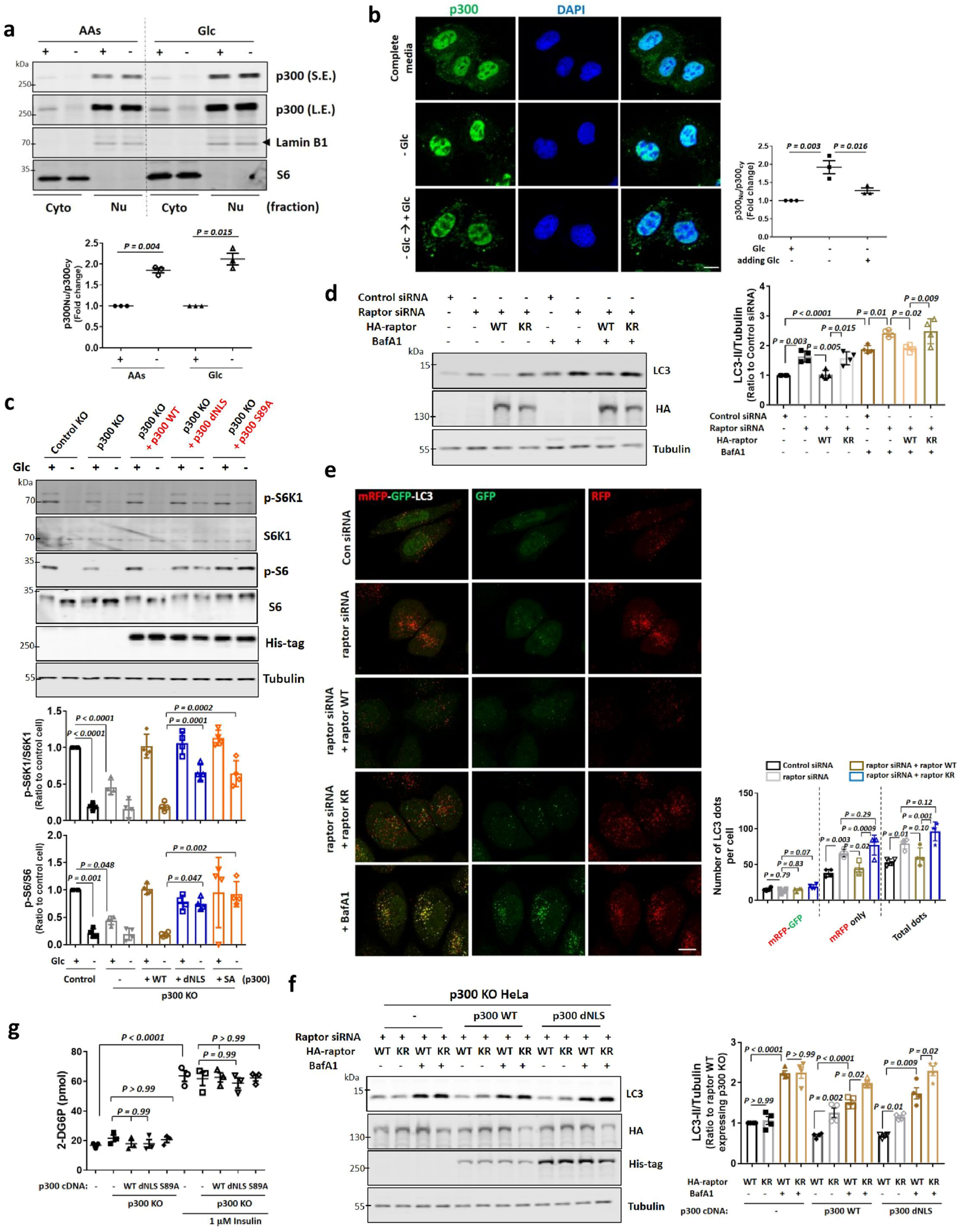

Extended Data Fig. 3
